# First initiation of mobilization out of bed after cardiac surgery – an observational cross-sectional study in Sweden

**DOI:** 10.1186/s13019-024-02915-4

**Published:** 2024-07-04

**Authors:** Elisabeth Westerdahl, Johanna Lilliecrona, Maria Sehlin, Anna Svensson-Raskh, Malin Nygren-Bonnier, Monika Fagevik Olsen

**Affiliations:** 1 University Health Care Research Center, Faculty of Medicine and Health, Örebro University, Örebro, Sweden; 2 Department of Health and Rehabilitation/Physiotherapy, Institute of Neuroscience and Physiology, Sahlgrenska Academy, University of Gothenburg, Gothenburg, Sweden; 3 Department of Physiotherapy, Sahlgrenska University Hospital, Gothenburg, Sweden; 4 Department of Community Medicine and Rehabilitation, Physiotherapy, Umeå University, Umeå, Sweden; 5 Division of Physiotherapy, Department of Neurobiology, Care Sciences and Society, Karolinska Institutet, Huddinge, Sweden; 6 Medical Unit Allied Health Professionals, Women’s Health and Allied Health Professionals Theme, Karolinska University Hospital, Stockholm, Sweden; 7 Department of Surgery, Institute of Clinical Sciences, Sahlgrenska Academy, University of Gothenburg, Gothenburg, Sweden

**Keywords:** Cardiac rehabilitation, Thoracic surgery, Physiotherapy, Postoperative care

## Abstract

**Background:**

Cardiac surgery is associated with a period of postoperative bed rest. Although early mobilization is a vital component of postoperative care, for preventing complications and enhancing physical recovery, there is limited data on routine practices and optimal strategies for early mobilization after cardiac surgery. The aim of the study was to define the timing for the first initiation of out of bed mobilization after cardiac surgery and to describe the type of mobilization performed.

**Methods:**

In this observational study, the first mobilization out of bed was studied in a subset of adult cardiac surgery patients (*n* = 290) from five of the eight university hospitals performing cardiothoracic surgery in Sweden. Over a five-week period, patients were evaluated for mobilization routines within the initial 24 h after cardiac surgery. Data on the timing of the first mobilization after the end of surgery, as well as the duration and type of mobilization, were documented. Additionally, information on patient characteristics, anesthesia, and surgery was collected.

**Results:**

A total of 277 patients (96%) were mobilized out of bed within the first 24 h, and 39% of these patients were mobilized within 6 h after surgery. The time to first mobilization after the end of surgery was 8.7 ± 5.5 h; median of 7.1 [4.5–13.1] hours, with no significant differences between coronary artery bypass grafting, valve surgery, aortic surgery or other procedures (*p* = 0.156). First mobilization session lasted 20 ± 41 min with median of 10 [1–11]. Various kinds of first-time mobilization, including sitting on the edge of the bed, standing, and sitting in a chair, were revealed. A moderate association was found between longer intubation time and later first mobilization (ρ = 0.487, *p* < 0.001). Additionally, there was a moderate correlation between the first timing of mobilization duration of the first mobilization session (ρ = 0.315, *p* < 0.001).

**Conclusions:**

This study demonstrates a median time to first mobilization out of bed of 7 h after cardiac surgery. A moderate correlation was observed between earlier timing of mobilization and shorter duration of the mobilization session. Future research should explore reasons for delayed mobilization and investigate whether earlier mobilization correlates with clinical benefits.

**Trial registration:**

FoU in VGR (Id 275,357) and Clinical Trials (NCT04729634).

**Supplementary Information:**

The online version contains supplementary material available at 10.1186/s13019-024-02915-4.

## Background

Early mobilization is a key component of postoperative care in cardiac surgery and includes physical activity and movement as soon as possible after surgery. Mobilization may involve changes in body position in bed, sitting up in bed or on the edge of the bed, standing by the edge of the bed, sitting in a chair/armchair, walking in the room/ward, cycle ergometer and climbing stairs [12–15]. Postoperative immobilization and sedation are well-known factors to the risk of postoperative complications and it is well known that assuming the supine position can impair the gas exchange in the lungs [1].

Nevertheless, it is not clear what is the best practice for mobilization after cardiac surgery [2]. Early mobilization protocols have been incorporated into the immediate postoperative period or first postoperative day, but a more detailed description of the exact timing has not been found in the literature. There are several benefits associated with early mobilization, including improved central and peripheral blood flow, prevention of vascular thrombosis and improved well-being [3, 4]. Changes in position from lying to sitting and to standing have a positive effect on lung volume and minute ventilation. Sitting in a chair 2–3 days following elective cardiac surgery has been shown to improve tidal volume, inspiratory capacity, alveolar ventilation and peripheral oxygen saturation (SpO_2_) [14].

Early mobilization is important for preventing complications, improving functional capacity, and shortening hospital stays for patients who have undergone major cardiac surgery [5]. Regardless of the type of mobilization, bed rest should be avoided, and early recovery should be facilitated. The time for initiating postoperative mobilization varies after surgery and can be performed even when patients are still on mechanical ventilation [4]. Mobilization is most often initiated during the day of surgery or the first postoperative day [6, 7]; however, studies on the exact time (hours) for the initiation of mobilization are limited [2, 8].

Encouraging patients to get out of bed early after surgery is most often incorporated into postoperative fast-track management following major cardiac surgery and is aimed at, minimizing postoperative morbidity, and enhancing overall recovery after surgery. Starting as early as possible based on the patient’s current status has been suggested to be preferable for counteracting the complications of immobilization [5].

The aim of the study was to define the timing for the first initiation of out-of-bed mobilization after cardiac surgery within the first 24 h after surgery and to describe the duration and type of mobilization performed during this first mobilization session. Additionally, potential associations between the timing of the first mobilization session and factors such as demographics, duration of intubation, and type of cardiac surgery were investigated.

## Methods

### Study design, setting and participants

The study design is an observational, cross-sectional, multicentre study investigating clinical practices regarding the first mobilization after cardiac surgery in Sweden. The study constitutes a subgroup analysis derived from the Survey of Mobilization and Breathing exercises After Thoracic and Abdominal surgery, the SOMBATA study [9]. In this subset, the first mobilization out of bed was examined in a cohort of cardiac surgery patients (*n* = 290) from five of the eight university hospitals performing cardiothoracic surgery in Sweden. The observational study period was the first 24 h after surgery.

The inclusion criteria were adult patients (> 18 years) undergoing elective, subacute or acute cardiac surgery with or without extracorporeal circulation (ECC) for coronary artery bypass grafting (CABG), valve replacement, valve repair, aneurysm, aortic dissection repair or combination surgery. Exclusion criteria were trauma or transplant surgery, or surgeries with an anaesthesia time < 2 h or single endovascular procedures. The study observational period was a consecutive five-week period (Monday–Thursday) between September 2021 and February 2022. The study was conducted as a survey of existing clinical mobilization routines at hospitals in Sweden during these five weeks. No sample size calculation for this substudy on cardiac surgery patients was performed. After bedside sampling, the collected data were completely deidentified and could not be linked to any of the patients during or after the analysis.

Cardiac surgery was performed under general anesthesia. Patients were given sedatives and analgesics as per standard routines at each respective hospital and were extubated after being transferred to the intensive care unit (ICU) or postoperative ward.

### Study performance and outcome variables

The timing and duration of the initial postoperative mobilization out of bed were observed and recorded for up to 24 h following surgery. The term “first mobilization” referred to the initial occasion of mobilization out of bed by at least moving from a supine position to sitting on the edge of the bed. Subsequently, this seated posture could advance gradually to standing by the bedside, sitting in a chair, stepping on the spot at the bedside or walking.

The physiotherapist assigned to the ward was designated as responsible for collecting descriptive, anaesthetic, and surgical data via medical records and surgery planning systems. Data collection started at the time of arrival at the first postoperative ward/ICU after surgery and was documented by nursing or physiotherapy staff using a study-specific registration protocol (see supplementary file 1). The outcome variables were time (hours) to the start of the first postoperative mobilization from (i) end of surgery; (ii) arrival at the first postoperative unit; and (iii) extubation. Additionally, the number and profession of assisting staff at the first mobilization session as well as the duration of the mobilization (in minutes), were noted. The support of health care staff (nurses, assistant nurses, or physiotherapists) in carrying out the mobilization was available to patients need.

### Statistical analysis

Descriptive demographic data are given as mean with standard deviation (SD), range (min-max), numbers and proportions or median with interquartile range [IQR]. The time intervals related to mobilization after the end of surgery, arrival at the postoperative ward, and extubation, as well as the duration of the first mobilization session are presented as the mean ± SD (range) and median [IQR] i.e. 1st – 3rd quartile. The Kruskal-Wallis test was performed to assess whether there were significant differences in time to first mobilization between the five surgery types (CABG, valve surgery, combination CABG and valve surgery, aortic surgery, and other procedures). A Mann-Whitney U test was performed to examine possible sex differences and the impact of daytime versus evening/night arrival on the time to first mobilization in the postoperative unit. Missing data was excluded from the analysis.

Spearman’s rank correlation coefficient (*ρ*) was used to assess possible correlations between patient age, BMI, intubation time or duration of the mobilization session, and time to first mobilization. In this study, a correlation coefficient of *ρ* = 0.10–0.29 was defined as weak, *ρ* = 0.30–0.49 as moderate, and *ρ* = 0.50–0.69 as strong, and *ρ* = 0.70–1.0 as very strong. A *p*-value < 0.05 was considered statistically significant. Statistical analyses were performed in IBM SPSS Statistics for Windows, version 25 (IBM Corp., Armonk, NY, USA.

## Results

**Fig. 1:**
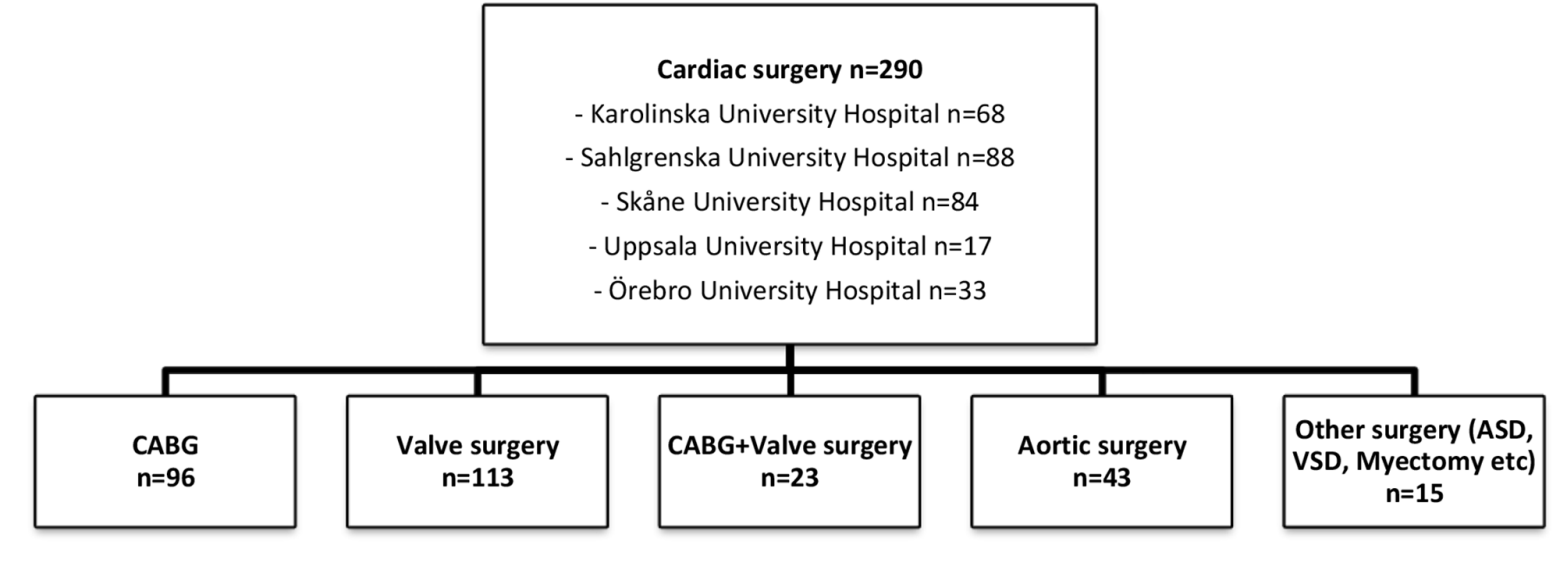
Flow chart of patients included in the study by type of surgery ASD = Atrial septal defect; CABG = Coronary artery bypass grafting; VSD = Ventricular septal defect

Patient characteristics and demographic data (*n* = 290) are summarized in Table 1. The five included university hospitals and types of surgeries are shown in Fig. 1. A total of 96% (*n* = 277) of the included patients were mobilized within 24 h after the end of surgery. The main reasons for not being mobilized within 24 h were: being intubated, being insufficiently awake/under sedation, or circulatory or respiratory instability (*n* = 13, 4%).

**Table 1:** Demographic characteristics of cardiac surgery patients (*n* = 290)

Variable		
Age, years		63 ± 14
Weight, kg		83 ± 17
BMI, kg/m^2^		27 ± 5
Sex, men, n (%)		226 (78%)
Active smoker, yes, n (%)		36 (12%)
Lung disease^a^, n (%)		30 (10%)
Overweight, BMI > 30 kg/m^2^ n (%)		70 (24%)
Immobilized, partly dependent/walking aids, n (%)		2 (1%)
ASA score, n (%)^b^	II	11 (3.5%)
	III	212 (75%)
	IV	59 (21%)
	V	1 (0.5%)
Anaesthesia time, h		5 ± 1
Surgery time, h		3 ± 2
Time of ECC, min		102 ± 50
Total intubation time, h		8 ± 3
Elective/subacute/acute surgery		71%/24%/5%
Full sternotomy/Minimally invasive		97%/3%
Perioperative bleeding, mL		496 ± 530

The data are presented as the mean (± SD) or n (%)

ASA = American Society of Anesthesiologists (physical status classification); BMI = body mass index; ECC = Extra corporeal circulation; SD = standard deviation

^a^) Lung disease = Obstructive or restrictive, ^b^) Missing values *n* = 7

**Fig. 2:**
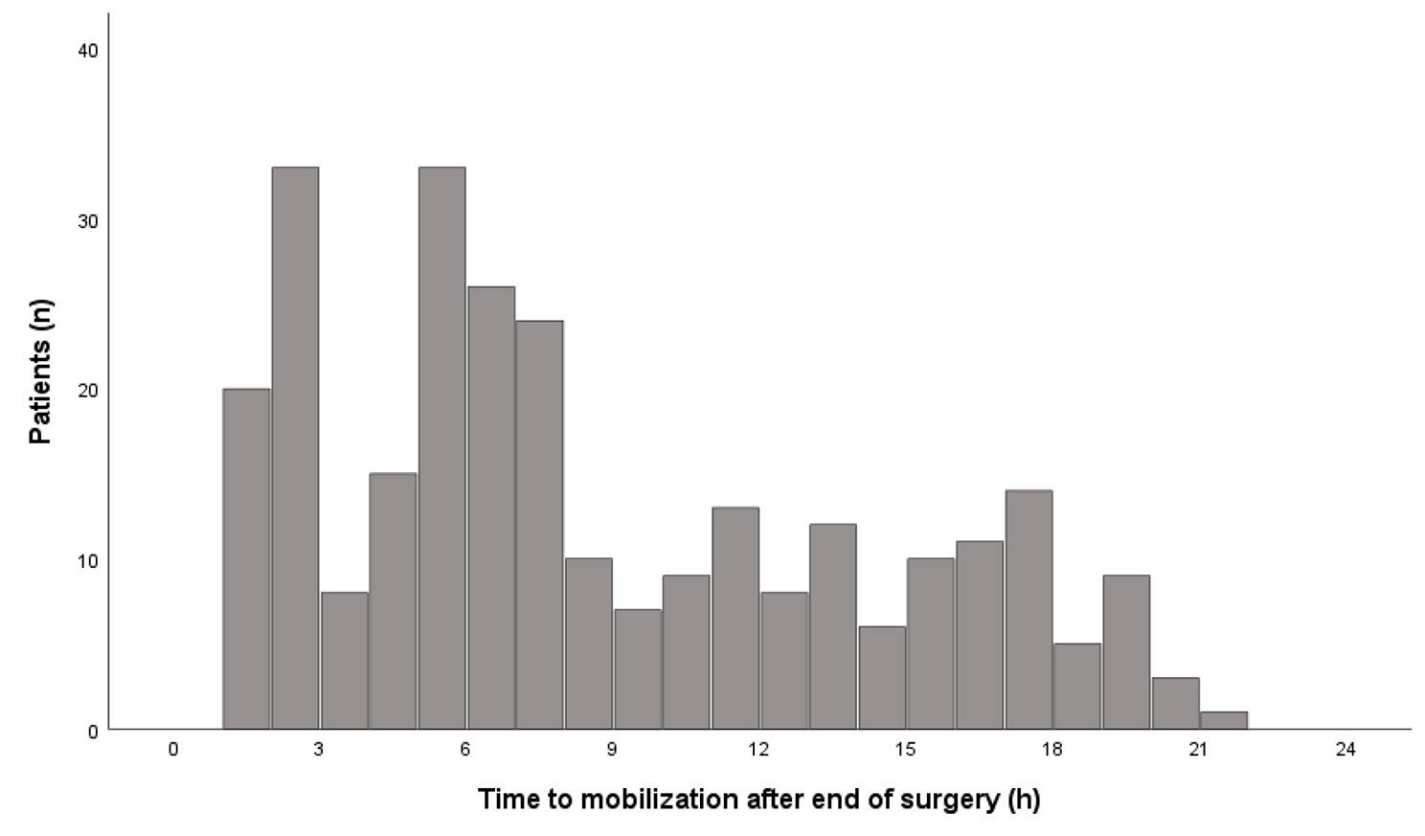
Time to first mobilization, defined as at least sitting on the edge of the bed, following cardiac surgery (*n* = 277)

Of the mobilized patients, 61% (*n* = 170) were mobilized on the day of surgery, and for the remaining 39% (*n* = 107) the first mobilization took place on postoperative day 1. The mean time to the first mobilization after the end of surgery was 8.7 ± 5.5 (range: 1–21) hours (*n* = 277) (Fig. 2). Time to mobilization after end of surgery; after arrival to the first postoperative unit, and from extubation for each surgical type are presented in Table 2.

**Table 2:** Time to first mobilization out of bed after the end of surgery, arrival at the postoperative unit, and after extubation, respectively, by type of surgery

Patients included	Mobilized within 24 h	Time (h) to first mobilization out of bed from:		
		end of surgery	arrival postop	extubation
All *n* = 290	*n* = 277	8.7 ± 5.5 7.1 [4.5–13.1]	8.2 ± 5.5 6.7 [4.0-12.6]	5.6 ± 4.7 3.9 [1.8-9.0]
CABG *n* = 96	*n* = 94	8.2 ± 5.6 6.6 [4.3–11.7]	7.8 ± 5.6 6.1 [3.9–11.4]	5.5 ± 5.0 3.2 [1.8–7.4]
Isolated valve *n* = 113	*n* = 110	8.2 ± 5.3 7.1 [3.6–12.3]	7.7 ± 5.3 6.7 [3.1–11.9]	5.4 ± 4.5 3.9 [1.7–8.3]
CABG and valve *n* = 23	*n* = 20	10.9 ± 5.0 10.5 [7.0-15.5]	10.3 ± 5.0 10.0 [6.7–14.9]	6.7 ± 4.0 6.2 [2.9–10.7]
Aortic surgery *n* = 43	*n* = 39	9.3 ± 5.7 7.1 [4.9–15.1]	8.9 ± 6.7 6.8 [4.6–14.6]	5.9 ± 4.8 3.9 [1.8–10.0]
Other procedures; ASD, VSD *n* = 15	*n* = 14	10.2 ± 5.8 8.5 [5.0-15.3]	9.8 ± 5.8 8.2 [4.8–14.8]	6.6 ± 5.7 4.0 [2.3–11.8]

Data as the mean ± SD and median [IQR].

ASD = Atrial septal defect; CABG = Coronary artery bypass grafting; IQR = Interquartile range; SD = standard deviation; VSD = Ventricular septal defect

The number of patients mobilized within 6 h after the end of surgery was (*n* = 109, 39%), 6–12 h after surgery (*n* = 90, 33%), and the remaining patients (*n* = 78, 28%) were mobilized 12–24 h after surgery.

**Fig. 3:**
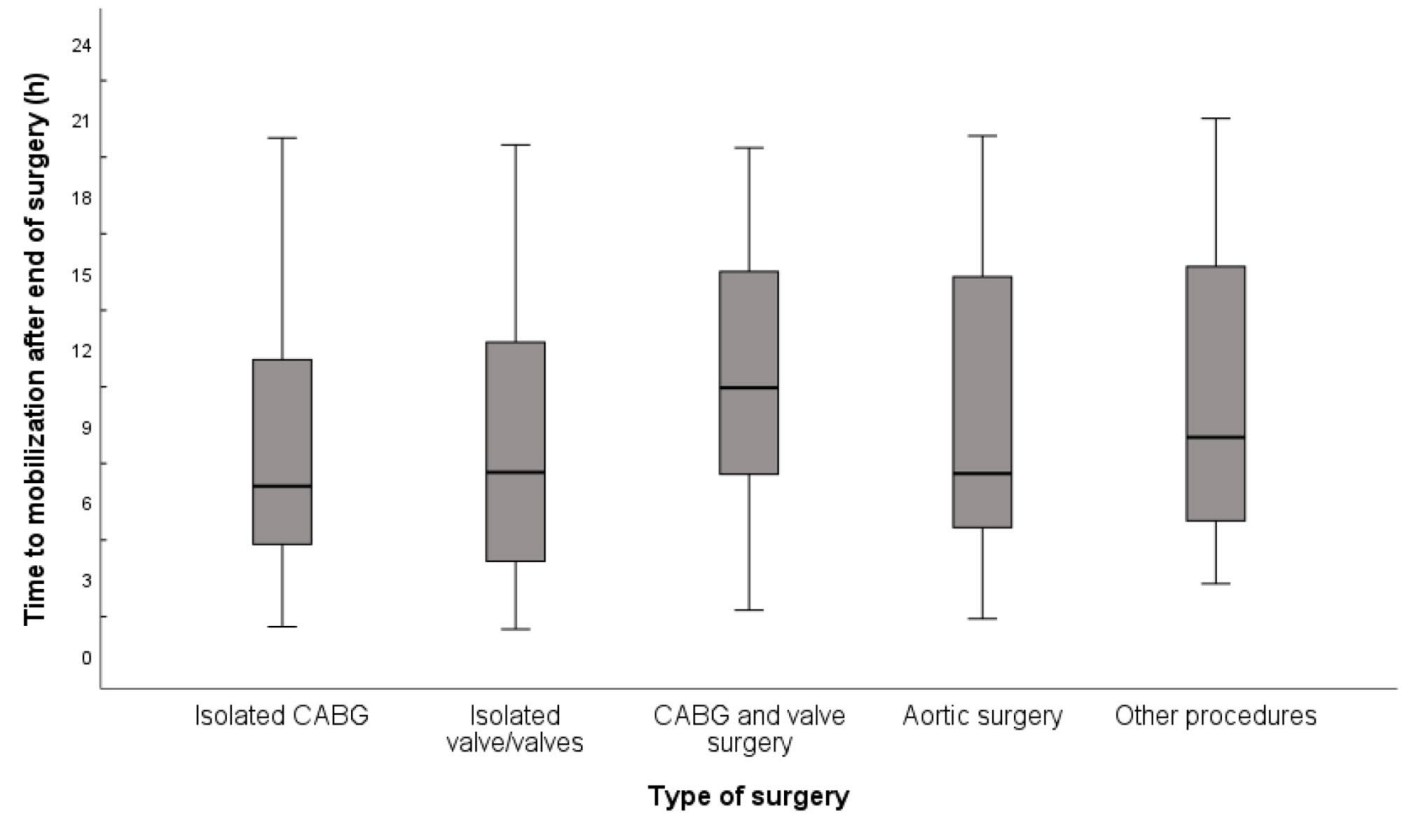
Time to first mobilization, defined as at least sitting on the edge of the bed, following cardiac surgery, categorized by type of surgical procedure (*n* = 277) The box-and whisker plots illustrate the interquartile ranhge [IQR] from the first quartile (Q1) to the third quartile (Q3), with the median line inside. Whiskers show the minimum and maximum values within 1.5 times the IQR CABG = Coronary artery bypass grafting; IQR = Interquartile range

There were no significant differences among the five surgical types (CABG, valve surgery, combination CABG and valve surgery, aortic surgery, and other procedures) regarding the time to first mobilization out of bed after the end of surgery (*p* = 0.156) (Fig. 3).

First mobilization was mostly performed at the postoperative ward/ICU by the nursing staff (*n* = 262, 90%). Patients arrived at the postoperative ward or intensive care unit mostly before 14:00 (*n* = 161, 58%), or in the afternoon, evening or night (*n* = 116, 42%). There was a significant shorter time to first mobilization in patients arriving at the postoperative unit before 14:00 than patients arriving in the afternoon, evening or night (*p* = 0.003).

In addition to being mobilized to sitting on the edge of the bed, 40% of the patients were also mobilized to standing, and 14% were mobilized to sit in a chair during the first mobilization out of bed. The type of mobilization performed during the first mobilization session are presented in Table 3.

**Table 3:** Type of mobilization performed at the first mobilization session after cardiac surgery (*n* = 290)

Type of mobilization	*n* (%)
Not mobilized within 24 h	13 (4.5%)
Sitting on the edge of the bed	31 (11%)
Sitting on the edge of the bed and standing	117 (40%)
Sitting on the edge of the bed, standing, and walking on the spot	88 (30%)
Sitting on the edge of the bed, standing, and sitting in a chair	40 (14%)
Sitting on the edge of bed, standing, sitting in a chair, and walking	1 (0.5%)

**Fig. 4:**
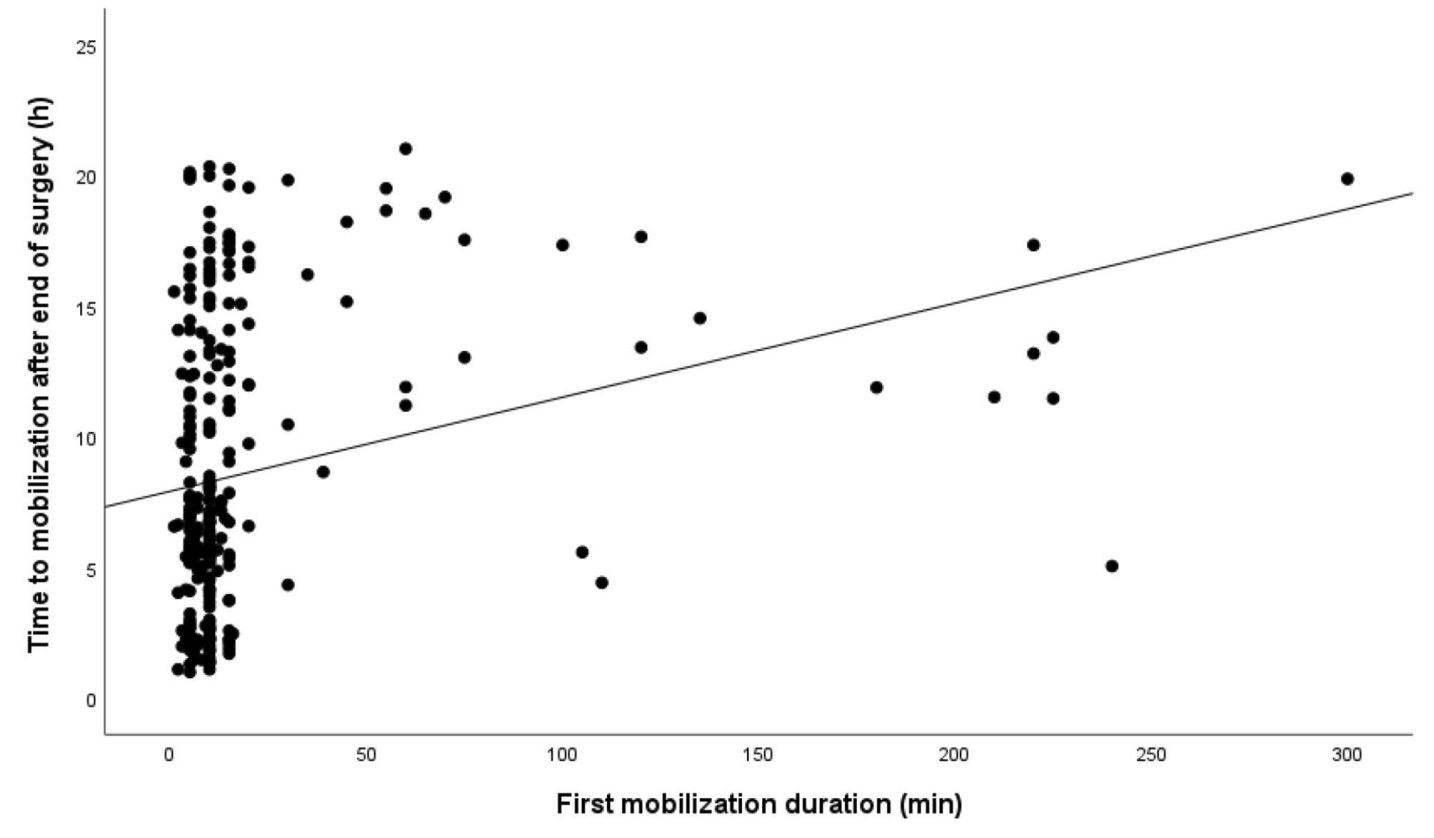
Time to first mobilization out of bed after cardiac surgery, by duration of first mobilization session (*n* = 277)

The mean duration of the first mobilization session was 20 ± 41 min (range: 1–300 min), with a median of 10 [1–11] minutes. In total, 66% (*n* = 191) of the patients had a mobilization time of less than 10 min, and 4% (*n* = 11) had a first mobilization session of more than 120 min. A moderate correlation was found between earlier mobilization and a shorter duration of the first mobilization session, with a Spearman’s rank correlation coefficient (ρ) of 0.315 (*p* < 0.001) (Fig. 4).

No significant correlations were revealed between age (*ρ* = 0.110, *p* = 0.068) or BMI (*ρ* -0.079, *p* = 0.193) and time to first mobilization out of bed after surgery. Regarding sex, there was no significant difference for commencement time of mobilization (*p* = 0.404). There was a moderate positive correlation between intubation time (*ρ* = 0.487, *p* < 0.001) and time to the first mobilization.

## Discussion

This study describes first postoperative mobilization routines in a cohort of 290 patients who underwent cardiac surgery in Sweden. Our investigation revealed that a majority (61%) of the patients were mobilized on the day of surgery, defined as mobilization to a sitting position on the edge of the bed. The median [IQR] time to first mobilization after the end of surgery was 7.1 [4.5–13.1] hours, with wide range (1–21 h). As expected, the mobilization time points after the end of surgery had the same range and were well matched with the arrival at the postoperative ward 6.7 [4.0-12.6] hours. Early mobilization is a cornerstone of postoperative treatment, even if routines may differ between hospitals. How early patients are actually getting out of bed after cardiac surgery has not previously been prospectively evaluated. Few studies on this topic are available. In a study by Ibrahimoglu et al., the median time for patients to first time sitting at the edge of the bed was 23 h after admission to the ICU [8]. In a study by Jacob et al., the mean time required for initiating the first mobilization out of bed were reduced from 23 h to 12 h after a quality improvement project aimed at enhancing early mobilization after cardiac surgery [2]. Our data demonstrated that 61% of patients had their first mobilization on the day of surgery. A small percentage (4%) of the total of 290 patients did not undergo mobilization within 24 h. The reasons for this delayed mobilization, included factors such as prolonged intubation, insufficient wakefulness or sedation, and circulatory or respiratory instability. Both patient-related and staff-related challenges are important to consider, and organizational adaptions may be needed, as concluded by Unver et al. [10]. Unfortunately, there are still only few studies describing the timing of first mobilization [2, 8]. Patients undergoing cardiac surgery under general anaesthesia develop atelectasis and impairments in pulmonary function in the early postoperative period [11]. Prolonged bed rest may be a risk factor for postoperative complications. The positive effects of a sitting position [14], standing position [16] and early mobilization protocol [17] in the early postoperative period after cardiac surgery have been demonstrated. Breathing exercises with the support of a physiotherapist may be provided during the mobilization and it is important that treatment is preceded by optimal pain relief. Several factors may hinder early initiation of mobilization, necessitating further studies on the safety and feasibility of enhancing early mobilization routines.

A considerable proportion of the included patients (20%) were mobilized within 0–3 h after the end of surgery. The average mobilization session lasted 20 ± 41 min, with a wide range between patients. Additionally, the type of mobilization varied, with 40% of patients mobilized to a standing position and 14% mobilized to sitting in a chair during their first session out of bed. These findings provide valuable insights into current practice for postoperative mobilization after cardiac surgery, emphasizing the promptness and variability of mobilization strategies, which could inform future clinical guidelines and improve patient care.

### Type of surgery

While early mobilization is generally beneficial, the timing and intensity of mobilization may vary based on the patient’s condition, the type of surgery performed, and other individual factors. Health care professionals, including nursing staff and physiotherapists, play a crucial role in assessing whether a patient is ready to be mobilized. The type of surgery was categorized according to the cardiac surgery national registry in Sweden, in which CABG accounts for approximately half of all heart surgeries [18]. No significant differences were observed between the five different types of surgery regarding the time to first mobilization out of bed (*p* = 0.173), although there appears to have been a trend that combined surgery patients were mobilized later than CABG patients (Fig. 3).

### Type of mobilization

In the first mobilization session following cardiac surgery, 40% of patients in this study engaged in sitting on the edge of the bed in combination with standing. Other common mobilization types included walking on the spot (30%) and sitting in a chair (14%). Mobilization, especially transitions such as shifting from sitting to standing, can induce fluctuations in gravitational forces, venous return, stroke volume and blood pressure [19]. Cardiac surgery patients are susceptible to various postoperative complications, such as arrythmias, decreased cardiac output, bleeding, and renal, pulmonary or neurological complications, and most often recover in the ICU during the initial postoperative period [20–22]. Therefore, adequate monitoring and well-trained health care staff are crucial for providing timely and appropriate care, ensuring patient safety, and preventing complications related to the mobilization. The ability to adjust mobilization based on cardiovascular stability and individual patient needs is crucial. Moreover, physical movement is recommended to be limited for up to 2–3 months after surgery, to enable time for the sternum to heal sufficiently. Adherence to proper mobilization technique and sternal precautions is essential. Patients are taught to get out of bed without stress of the sternum and various movement protocols are being developed [23, 24]. A variety of physical activities, such as early bed activities, cycle ergometers, exercise, and walking protocols, are prescribed after cardiac surgery [15, 25, 26]. Early mobilization is often combined with respiratory exercise, psychoeducation and aerobic training, and these protocols have shown improvements in 6-minute walking tests at hospital discharge [27], better sleep, shorter hospitalization and fewer late complications [6]. Early mobilization and exercise are prescribed to prevent complications and shorten the hospital stay, but there is still a lack of evidence on effectiveness [5, 28, 29]. Different studies use different categorization of mobilization activities, potentially making direct comparisons difficult. After discharge from the hospital, an association between increased physical activity and recovery of lung function has been shown in cardiac surgery patients [30]. The variability in mobilization practices, underscoring the need to investigate the factors affecting mobilization timing and level and their potential impact on patient recovery.

### Associations with timing of initial mobilization

A correlation between length of intubation and time to first mobilization was found in accordance with results of Ibrahimoglu et al. [8]. Early extubation and encouraging an alert patient to be active can support timely mobilization and contribute to fostering enhanced recovery protocols after cardiac surgery, however, it is essential to balance benefits and risks [31, 32]. Our findings also suggest a weak positive correlation between time to mobilization and duration of mobilization, indicating that as the time to mobilization increases, there is a tendency for the duration of mobilization to increase. This could be important to consider when planning activities during the first mobilization session.

### Study limitations

There are some study limitations. The study involved a sample of 290 patients from only five out of eight university hospitals performing cardiothoracic surgery in Sweden, potentially limiting the generalizability of the results. The study was conducted during the pandemic, which led to challenges with healthcare staffing, this might have influenced who was available and willing to participate in the study, potentially leading to a selection bias. This study focused on the first 24 h after surgery, which failing to capture variations in mobilization practices beyond this timeframe. Examining a more extended period might provide a more comprehensive understanding of postoperative mobilization. The prospective study design, however, offered robust and real-time data collection, reducing potential recall bias associated with retrospective data collection, which could have strengthened the accuracy of data. The healthcare staff involved were informed about the study, which might have influenced and stimulated mobilization activities, possibly due to the Hawthorne effect. Using wearable accelerometers [33] could have provided a more precise and quantifiable description of mobilization, potentially enhancing the reliability and strengthening the findings of this study.

## Conclusion

In conclusion, our study provides the first detailed overview of initial mobilization following cardiac surgery in Sweden. The median time of 7 h to first mobilization out of bed demonstrates a strong commitment to prompt mobilization after surgery.

Notably, a substantial proportion (39%) were mobilized within the initial 6 h after surgery. However, it was found that earlier timing of mobilization was associated with a shorter duration of the mobilization session. No significant differences were noted in the time to first mobilization out of bed between different surgery types.

These findings contribute valuable insights into the current practices of postoperative mobilization after cardiac surgery. The results may serve as a basis for refining clinical protocols and enhancing patient-centred care in the postoperative period. Further research is warranted to explore the optimal timing and duration of early mobilization interventions to maximize benefits while minimizing risks.

### Electronic supplementary material

Below is the link to the electronic supplementary material.

Supplementary Material 1

## Data Availability

Data and analytical methods will be shared on reasonable request.
