# Supplementary material for: First initiation of mobilization out of bed after cardiac surgery – an observational cross-sectional study in Sweden
Source: J Cardiothorac Surg. 2024 Jul 4;19:420. doi: 10.1186/s13019-024-02915-4 (PMC11223441; doi:10.1186/s13019-024-02915-4)
Supplement: Supplementary file 1 — Supplementary Material 1 [file 13019_2024_2915_MOESM1_ESM.pdf]

Survey Of Mobilisation and Breathing exercises After Thoracic and Abdominal surgery

Hospital	
Date of surgery	

Following data is registered within the first 24 hours after surgery

**Arrival to the first postoperative unit (after surgery)**	
Time	

**The first mobilisation after surgery**					
Start, time:		Back in bed, time:			
Sitting on the edge of the bed ☐	Standing ☐	Sitting in a chair ☐	Walking by the bed ☐	Walking ☐ ___ m	
Assistanced by	Nurse ☐	Assistant nurse ☐	Physiotherapist ☐	Other ☐	
Assistence of number or staff:		1 ☐ 2 ☐	3☐	4 ☐	
Performed where		ICU/postop ☐	Intermedian ward ☐	Surgical ward ☐	

No mobilisation performed within the first 24 hours because of:		
Sedated ☐	Circulatory instability ☐	Respiratory instability ☐
Not prioritized ☐	Time constraint ☐	Lack of staff ☐
Other reason ☐ definie________________________		

**Start of specific breathing exercises after surgery**		
Start, time		
Deep breathing exercises ☐	Incentive Spirometry ☐	Respiratry Muscle Training ☐
Bubble-PEP ☐	PEP-mask ☐	PEP-by mouth-piece ☐
Other ☐ describe		
Specific breathing exercises prescribed but not performed ☐		No breathing exercises necessary ☐

**At discharge to the surgical ward**	**Not discharged during the first 24 hrs ☐**
Discharged when, time _______________	Day of surgery ☐ Day after surgery ☐
Oxygen supply, No ☐ yes ☐, ___ litre or _______ %	SpO_2 (with oxygen supply) __________%

Following are registered by responsible of the trial

Background data					
	Age, years		Weight, kg	Height, cm	
Sex	Male ☐		Female ☐		
High risk factors	None ☐		Yes ☐, mark factors below		
	□ Pulmonary disease		☐ Obesity BMI>30 kg/m²		
	☐ Smoker		☐ Old age (>70 years)		
	☐ Immobilized		☐ Other, define		
Independent in movements preop	No ☐		Yes ☐		
Walking aids	No ☐		Yes ☐, what		
Preop information by PT	No ☐ Reason:		Yes, oral ☐ written ☐ film ☐		
Preop exercises	No ☐		Yes ☐ what:		
ASA-score	1 ☐	2 ☐	3 ☐	4 ☐	5☐
Procedure/anaethesia					
Anaesthesia, duration	Started		Finished		
Procedure, duration	Started		Finished		
Extubation, time	Tine		Not extubated during the first 24 h ☐		
Planned surgery					
Performed surgery					
Kind of procedure	Open ☐		Scopic ☐	Scopic with robot ☐	
	Elective ☐		Acute ☐	Subacute ☐	
Anaesthesia	Intravenous ☐ Gas ☐ (T)EDA ☐ Spinal ☐				
Heartlung mashine	No ☐		Yes ☐ Duration min:____________		
Perioperative bleeding	ml				
Analgesia postoperatively	Epidural ☐		Spinal ☐	Local ☐	
	Per os ☐		Intravenous ☐		
Fast track concept	No ☐	Yes ☐ ERAS ☐ ERP ☐ Fast-track □ Other ☐			

**Additional breathing supporting during the first 24 postoperative hours**			
CPAP ☐	Bilevel PAP ☐	High flow oxygen ☐	Cough assist ☐
Other ☐ Describe __________			
No additional treatments given ☐			
